# Low Crude Protein Diet Affects the Intestinal Microbiome and Metabolome Differently in Barrows and Gilts

**DOI:** 10.3389/fmicb.2021.717727

**Published:** 2021-08-20

**Authors:** Xin Tao, Bo Deng, Qizhi Yuan, Xiaoming Men, Jie Wu, Ziwei Xu

**Affiliations:** Institute of Animal Husbandry and Veterinary Science, Zhejiang Academy of Agricultural Sciences, Hangzhou, China

**Keywords:** dietary protein level, growing-finishing pig, microbiota, metabolite, barrow, gilt

## Abstract

Low protein diets are commonly used in the growing-finishing pig stage of swine production; however, the effects of low dietary protein on the intestinal microbiota and their metabolites, and their association with pig sex, remain unclear. The present study aimed to assess the impact of a low crude protein (CP) diet on the gut microbiome and metabolome, and to reveal any relationship with sex. Barrows and gilts (both *n* = 24; initial body = 68.33 ± 0.881 kg) were allocated into two treatments according to sex. The four groups comprised two pairs of gilts and barrows fed with a high protein diet (CP 17% at stage I; CP 13% at stage II) and a low protein diet (CP 15% at stage I; CP 11% at stage II), respectively, for 51 d. Eight pigs in each group were slaughtered and their colon contents were collected. Intestinal microbiota and their metabolites were assessed using 16S rRNA sequencing and tandem mass spectrometry, respectively. The low protein diet increased intestinal microbiota species and richness indices (*P* < 0.05) in both sexes compared with the high protein diet. The sample Shannon index was different (*P* < 0.01) between barrows and gilts. At the genus level, unidentified *Clostridiales* (*P* < 0.05), *Neisseria* (*P* < 0.05), unidentified *Prevotellaceae* (*P* < 0.01) and *Gracilibacteria* (*P* < 0.05) were affected by dietary protein levels. The relative abundance of unidentified *Prevotellaceae* was different (*P* < 0.01) between barrows and gilts. The influence of dietary protein levels on *Neisseria* (*P* < 0.05), unidentified *Prevotellaceae* (*P* < 0.01) and *Gracilibacteria* (*P* < 0.05) were associated with sex. Metabolomic profiling indicated that dietary protein levels mainly affected intestinal metabolites in gilts rather than barrows. A total of 434 differentially abundant metabolites were identified in gilts fed the two protein diets. Correlation analysis identified that six differentially abundant microbiota communities were closely associated with twelve metabolites that were enriched for amino acids, inflammation, immune, and disease-related metabolic pathways. These results suggested that decreasing dietary protein contents changed the intestinal microbiota in growing-finishing pigs, which selectively affected the intestinal metabolite profiles in gilts.

## Introduction

In swine production, normal dietary crude protein (CP) concentration is about 15–22%. However, reducing dietary protein levels has marked benefits, including increasing nitrogen (N) utilization, lessening seasonally feed costs, especially reducing the excretion of N into the environment. Although the recommendation for the dietary CP content was replaced with total N in the new edition of the NRC guidelines [National Research Council (NRC), 2012]. CP% could be calculated by multiplying the common CP coefficient of 6.25, thus dietary CP content recommended by NRC (2012) is reckoned decreasing by 2–4% than the previous edition NRC (1998) [National Research Council (NRC), 1998]. Furthermore, with the development of industrial synthetic amino acids (AA) technology, low protein diet strategy has become increasingly popular, especially in growing-finishing pigs, because pigs in this stage have very large populations, gain the most weight, consume large amounts of dietary protein, and excrete the maximum amount of nitrogen as slurry. Studies in piglets showed that dietary protein levels altered the intestinal microbiota compositions and microbially-derived metabolites (Rist et al., 2013). In growing-finishing pigs, because of their relatively stable microbiota structure, studies focused on environmental influences of N extraction (Zhao et al., 2019; Kim et al., 2020; Wang Y. et al., 2020). In fact, increasing studies indicate that the compositions and structure of the intestinal microbiota can change dynamically in response to many factors, including feed (Isaacson and Kim, 2012; Ananthakrishnan, 2015). Even in older life, the gut microbiota has the propensity of accelerating compositional change (Gi Langille et al., 2014). More significantly, sex-specific differences of intestinal microbiota due to sex hormones in human have been reported (de la Cuesta-Zuluaga et al., 2019). Therefore, we are interested in whether the impacts of dietary nutrients on intestine microbiota and metabolites are associated with pig sex. This is very important for precision nutrition of sex-specific feeding measures.

The mammalian gastrointestinal tract is colonized by thousands of microbial strains that form highly dense, dynamically changing, and extremely complicated communities exceeding 100 trillion microbial cells (Collins et al., 2012). These intestinal microorganisms, which metabolize dietary substances to obtain nutrients and energy while producing other metabolites, play vital roles in host physiology and nutrient metabolism (Lee and Hase, 2014). Therefore, dietary nutrients containing proteins, lipids, and carbohydrates decide the available substrates for the host intestinal microbiota, and affect microbial populations and their metabolic activities. Alternatively, the intestinal microbiota and their metabolic substances that are changed by dietary nutrients could further affect host health and physiological function (Shibata et al., 2017). In particular, intestinal microbial-derived metabolites have been reported recently to control gut inflammatory responses (Arpaia and Rudensky, 2014), thus directly influencing animal body health (Flint, 2016; Glowacki and Martens, 2020), and regulating host immunity (Kim et al., 2016). Therefore, revealing the relationship among dietary nutrients and intestinal microbiota and metabolites is important to improve the health and growth of mammals by regulating dietary compositions.

Protein is an essential nutritional component in human and animal diets throughout life processes. However, undigested excess dietary protein and amino acid will remain in the large intestine, and be fermented by abundant microbes, and generate some metabolites such as ammonia, hydrogen sulfide, and phenolic compounds which will have toxic influences on the host (Libao-Mercado et al., 2009; Kårlund et al., 2019). The microbial fermentation-derived metabolites from proteins are increasing recognized to induce the gut inflammatory response, increase intestinal permeability, and colitis, and are involved in the development of certain metabolic diseases and colorectal cancer (Diether Natalie and Willing Benjamin, 2019). Although both the contents and sources of protein have been demonstrated to change the gut microbiota and their metabolites (Lang et al., 2018), the protein contents, rather than sources, have a much greater impact (Rist et al., 2013). Long-term excessive protein intake has been shown to shift the microbiota composition, and change microbial taxa and fermentation pathways (Korpela, 2018). A low protein diet or dietary protein consumption with high digestibility could reduce the amount of protein reaching the colon, thus limiting the available protein for fermenting bacteria (Mayneris-Perxachs et al., 2016; Ma et al., 2017). However, there have been inconsistent conclusions about the influence of dietary protein levels on the intestinal microbiota. Some studies have reported that moderate dietary protein restriction could optimize the gut microbiota composition to some extent (Chen X. et al., 2018; Liu et al., 2021), while others have suggested that low protein diet decreased the gut microbiota diversity (Singh et al., 2017; Masuoka et al., 2020).

In view of the foregoing, we hypothesized that low protein diets could change the profile of intestinal microbiome and metabolome in the growing-finishing pigs. And these influences may be different in sexes. In the present study, the feeding experiment on barrows and gilts separately fed high protein diet and low protein diet was performed to test this hypothesis.

## Materials and Methods

### Animals and Experimental Treatments

A total of 24 barrows and 24 gilts (Duroc × Landrace × Yorkshire, 120 ± 2 days old) in the growing-finishing stage with an average initial body weight of 68.33 ± 0.881 kg were randomly assigned to one of two dietary treatments by sex (*n* = 12). Therefore, the following four groups in this experiment were treated: (1) BHP, barrows fed a high protein diet containing 17% CP in stage I and 15% CP in stage II; (2) GHP: gilts fed a high protein diet containing 17% CP at stage I and 15% CP at stage II; (3) BLP, barrows fed a low protein diet containing 13% CP at stage I and 11% CP at stage II; and (4) GLP, gilts fed a low protein diet containing 13% CP at stage I and 11% CP at stage II. Feed intake and body weight of every experimental animal were recorded using OSBORNE FIRE^®^ performance testing systems Osborne Industries, Inc., Osborne, KS, United States) which include feed intake and performance testing equipment, and automated growth management systems which could perform automatic weighing by recognizing the electronic ear mark during the feed eating time. Therefore, each treatment had one pen with 12 pigs. The experimental diets were formulated based on corn-soybean meal and met the nutritional needs of growing-finishing pigs according to the National Research Council [National Research Council (NRC), 2012] (Supplementary Table 1). All pigs had *ad libitum* access to food and clean drinking water. The experiment lasted 51 days, and the stage I was 32 days and stage II was 19 days.

### Sample Collection and Preparation

The feeding trial ended up while the average final body weight of all experimental pigs was 105.8 ± 1.228 kg. Eight pigs that were the closest to the average body weight of those in their treatment group were selected and electronically stunned and exsanguinated. The gastrointestinal tract was removed after the pigs’ abdominal cavities were opened. A colonic content sample of each pig, which has the most abundant microbiota populations in all intestinal sections and where protein fermentation is thought to mostly occur in the region (Williams et al., 2001; Wang Y. et al., 2020), was collected in 5 mL sterile frozen tubes, immediately flash-frozen in liquid nitrogen, and stored at -80°C for further analysis.

### 16S rRNA Amplicon Sequencing

Microbial genomic DNA from colonic content samples was extracted using the cetyltrimethylammonium bromide (CATB)/sodium dodecyl sulfate (SDS) method. The DNA concentration and purity of all DNA samples was estimated using a NanoDrop1000 spectrophotometer (Nanodrop Technologies, Wilmington, DE, United States) and 1% agarose gel electrophoresis. According to the concentration, DNA was diluted to 1 ng/μL using sterile water.

Amplicons were generated by PCR of the hypervariable region V3-V4 (341F-806R) of the bacterial 16S rRNA gene using Phusion^®^ High-Fidelity PCR Master Mix with GC Buffer (NEB, Ipswich, MA, United States). The obtained PCR products were purified using GeneJET kit (Thermo Scientific, Waltham, MA, United States). The purified products were used to construct a DNA library using Ion Plus Fragment Library Kit 48 rxns (Thermo Scientific). Sequencing was performed on a Thermo Fisher Ion S5TMXL platform (Thermo Scientific).

### Untargeted Metabolomics Analysis

Colon content samples were homogenized in prechilled methanol and 0.1% formic acid by vortexing. The homogenates were incubated on ice for 5 min and then centrifuged at 5,000 × *g*, at 4°C for 5 min. The supernatant samples were diluted to final concentration 60% aqueous methanol. Subsequently, the samples were transferred to Eppendorf tubes with 0.22 μm filters and were centrifuged at 5,000 × *g*, at 4°C for 10 min. Finally, the filtrates were injected into the liquid chromatography with tandem mass spectrometry (LC-MS-MS) system for analysis.

LC-MS/MS analyses were performed using an ultra-high performance liquid chromatography (UHPLC) system (Thermo Scientific) coupled with an Orbitrap Q Exactive HF-X mass spectrometer (Thermo Scientific). Samples were injected onto a Hyperil Gold column (100 × 2.1 mm, 1.9 μm) using a 16 min linear gradient at a flow rate of 0.2 mL/min. The eluents for the positive polarity mode were eluent A (0.1% formic acid in Water) and eluent B (methanol). The eluents for the negative polarity mode were eluent A (5 mM ammonium acetate, pH 9.0) and eluent B (methanol). The solvent gradient was set as follows: 2% B, 1.5 min; 2–100% B, 12.0 min; 100% B, 14.0 min; 100–2% B, 14.1 min; 2% B, 16 min. A Q Exactive HF-X mass spectrometer was operated in positive/negative polarity mode with spray voltage of 3.2 kV, a capillary temperature of 320 °C, a sheath gas flow rate of 35 arb, and an aux gas flow rate of 10 arb.

### Data Processing

Microbiome. Raw data were processed according to the Cuadapt (Martin, 2011) quality control process. Then the reads were compared with the reference database (Quast et al., 2012) using the UCHIME algorithm (Knight, 2011) to detect chimeric sequences, which were removed (Haas et al., 2011). Finally, clean data were obtained and used for subsequent analysis. Sequence analysis was performed by Uparse software version 7.0.1001 according to a previously published method (Edgar, 2013). Sequences with ≥ 97% similarity were assigned to the same operational taxonomic units (OTUs). OTU abundance information was normalized using a standard of the sequence number corresponding to the sample with the least sequences. Analyses of alpha diversity (α-diversity) and unweighted pair-group method with arithmetic mean (UPGMA) cluster analysis were performed based on the normalized data. Five indices including, observed species, Simpson index, Shannon index, Chao1 and ACE were calculated using QIIME Version1.7.0 (Caporaso et al., 2010). The top 10 most abundant communities at the phylum and genus levels were defined as predominant bacteria and compared among different treatments.

Metabolomics. The raw data files generated by UHPLC-MS/MS were processed using the Compound Discoverer 3.0 (CD 3.0, Thermo Scientific) to perform peak alignment, peak picking, and quantitation for each metabolite. The main parameters were set as follows: retention time tolerance, 0.2 min; actual mass tolerance, 5 ppm; signal intensity tolerance, 30%; signal/noise ratio, 3; and minimum intensity, 100000. Next, the peak intensities were normalized to the total spectral intensity. The normalized data were used to predict the molecular formula based on additive ions, molecular ion peaks, and fragment ions. The peaks were then matched with the mzCloud^1^ and ChemSpider^2^ databases to obtain the accurate qualitative and relative quantitative results for principal components analysis (PCA) and partial least squares discriminant analysis (PLS-DA). Based on the differential metabolites identified by comparing GHP and GLP, Kyoto Encyclopedia of Genes and Genomes (KEGG) pathway^3^ analysis was conducted to investigate the metabolomics pathways affected by protein level.

### Statistical Analysis

Statistical analysis was performed using the General Linear Model (GLM) procedures in SPSS V.19.0 (IBM Corp., Armonk, NY, United States) for a 2 × 2 factorial arrangement of treatments. The statistical model consisted of the fixed effects of dietary protein levels and pig sexes and their interactions. Single comparison between GHP and GLP while different abundant metabolites were analyzed was performed using Student’s *t* test in SPSS. The correlation between significantly changed bacteria (at the genus level) and metabolites in GHP and GLP were analyzed by Spearman’s rank correlation test using GraphPad Prim V.8.0 (GraphPad Software, San Diego, CA, United States). The results were expressed as mean and SEM. The *P* < 0.05 was considered significant. All the indices were analyzed with pig as the experimental unit (*n* = 8).

## Results

### DNA Sequence Coverage in Colonic Content

As shown in Supplementary Table 2, 2,668,457 V3-V4 16S rRNA valid sequences reads were obtained from 32 samples, including 78,848 (GHP), 88,485 (GLP), 85,353 (BHP), and 80,871 (BLP) raw reads. After removing chimeric sequences, 74,565, 82,306, 80,145, and 75,997 clean reads remained in the GHP, GLP, BHP, and BLP groups, respectively. The effective proportion of clean reads was 90.14–97.79%. The GC% among the clean reads was 70.16–82.57%. The results showed that there were no dramatic differences in the number of clean reads among the groups.

**FIGURE 1:**
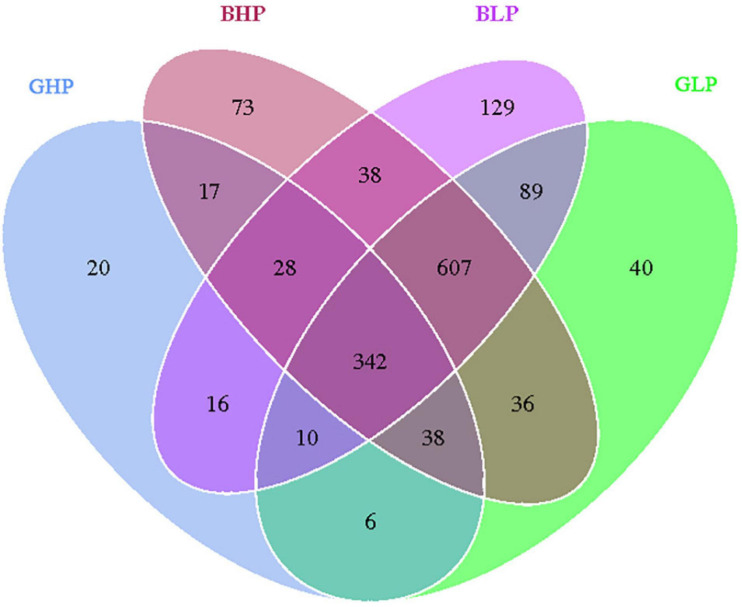
Venn diagram showing the unique and shared OTUs in the different groups. GHP, gilts fed high protein diet; BHP, barrows fed high protein diet; GLP, gilts fed low protein diet; BLP, barrows fed low protein diet.

The Venn diagram analysis of OTUs is shown in Figure 1. There were 342 common OTUs among all groups, and 20, 40, 73, and 129 unique OTUs were identified in the GHP, GLP, BHP, and BLP groups, respectively. From the perspective of dietary crude protein levels, there were 396 common OTUs in the gilt groups, 81 unique OTUs in the GHP group, and 772 in the GLP group. Similarly, there were 1,015 common OTUs in barrow groups, and 164 unique OTUs in the BHP group, and 244 in the BLP group. These results suggested that there were fewer common OTUs in gilts than in barrows. With respect to pig sex, there were 425 common OTUs in the HP groups, with 804 unique OTUs in the BHP group, and 90 in the GHP group. Similarly, there were 1,060 common OTUs in the LP groups, with 264 unique OTUs in the BLP group, and 158 in the GLP group. The results suggested that there were fewer common OTUs in the HP groups than in the LP groups.

### Observed Species, Microbial α-Diversity, and Cluster Analysis

As shown in Table 1, the low protein diet increased the number of observed species significantly compared with high protein diet (*P* < 0.05). In contrast, pig sex had no significant effects (*P* > 0.05). However, pig sex, rather than dietary protein levels, affected the Shannon index significantly (*P* < 0.01) and was higher in barrows than in gilts. Neither the dietary protein levels nor sex affected the Simpson index. Sample richness indices (ACE and Chao1) were higher (*P* < 0.05) in the pigs fed the low protein diet. Both indices showed no remarkable differences (*P* > 0.05) between gilts and barrows. In terms of all the α-diversity indices, no significant interactions (*P* > 0.05) were found between dietary protein levels and pig sex.

**TABLE 1:** Number of observed species, richness and Alpha diversity indices in the colon content samples from each group.

Index	Treatment				SEM^5^	*P*-value		
	GHP^1^	BHP^2^	GLP^3^	BLP^4^		CP^6^	Sex^7^	CP × Sex
Observed species	309.8	455.0	491.1	516.5	26.59	0.029*	0.118	0.267
Shannon	5.320	6.022	5.724	6.239	0.099	0.133	0.005**	0.644
Simpson	0.937	0.951	0.938	0.965	0.005	0.499	0.078	0.557
Chao1	358.7	526.6	583.5	599.5	30.89	0.022*	0.144	0.225
ACE	364.8	540.4	600.3	605.2	31.21	0.021*	0.153	0.176

*Data are means of eight observations.*

*^1–4^GHP, gilts fed high protein diet; BHP, barrows fed high protein diet; GLP, gilts fed low protein diet; BLP, barrows fed low protein diet.*

*^5^SEM, standard error of the mean.*

*^6^CP: crude protein levels, for high protein level and low protein level.*

^7^Sex: for gilts and barrows. *Statistically significant (*P* < 0.05); ** statistically very significant (*P* < 0.01).

**FIGURE 2:**
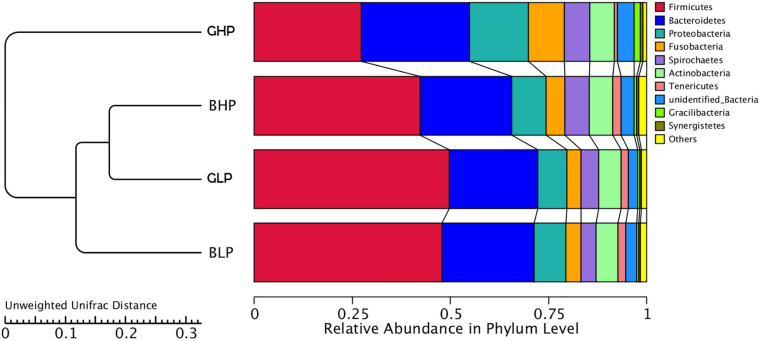
UPGMA cluster tree based on weighted unifrac distances of the OTU community. GHP, gilts fed high protein diet; BHP, barrows fed high protein diet; GLP, gilts fed low protein diet; BLP, barrows fed low protein diet.

As shown in Figure 2, the UPGMA cluster of community structures at the phylum level were analyzed among the four treatments. The results showed that the GLP and BLP groups were the closest, and then they clustered together with BHP, followed by GHP, indicating that dietary protein levels had greater effects on microbial community structures than pig sex.

### Relative Abundance of the Predominant Microbial Community Induced by Dietary Protein Levels in the Colonic Content of Barrows and Gilts

**FIGURE 3:**
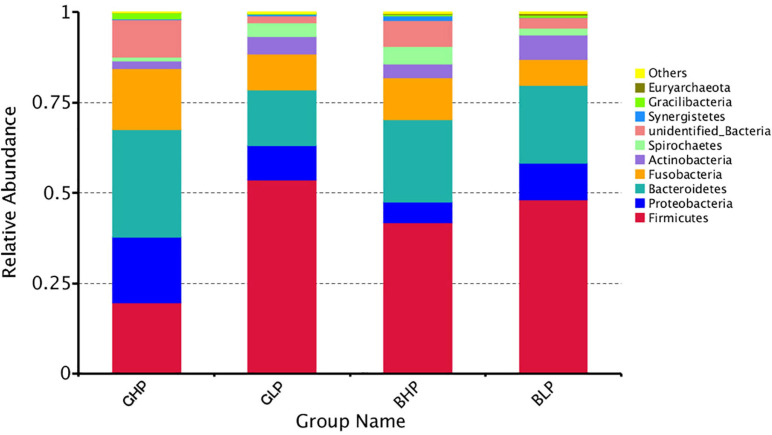
Relative abundance of microbial community (Top 10) structure in the colonic content at Genus level. GHP, gilts fed high protein diet; BHP, barrows fed high protein diet; GLP, gilts fed low protein diet; BLP, barrows fed low protein diet.

The results for the relative abundance of top 10 members of the microbial community structure in the colonic contents at different levels (phylum and genus) were analyzed. At the phylum level (Supplementary Figure 1 and Table 2), reducing dietary protein level significantly increased the abundance of *Actinobacteria* (*P* < 0.05) and decreased the abundance of unidentified bacteria (*P* < 0.01). No remarkable differences (*P* > 0.05) in the abundance of any microbial community were found between gilts and barrows. For *Proteobacteria*, *Gracilibacteria*, and *Synergistetes*, there were significant interactions (*P* < 0.05) between dietary protein levels and pig sex. At the genus level (Figure 3 and Table 3), the top three microbial community, including unidentified *Clostridiales* (*P* < 0.05), *Neisseria* (*P* < 0.05), and unidentified *Prevotellaceae* (*P* = 0.00) were significantly affected by dietary protein levels. The abundance of unidentified *Prevotellaceae* was also different (*P* < 0.01) between gilts and barrows. Dietary protein levels and pig sex showed significant interactions in *Neisseria* (*P* < 0.05) and unidentified *Prevotellaceae* (*P* = 0.00). In addition, the abundances of *Gracilibacteria* (*P* < 0.05) and unidentified bacteria (*P* = 0.00) were significantly affected by dietary protein levels, and showed significant interactions (*P* < 0.05) between dietary protein levels and pig sex.

**TABLE 2:** Relative abundance of microbial community (Top 10, %) structure in colonic content at phylum level from each group.

Index	Treatment				SEM^5^	*P*-value		
	GHP^1^	BHP^2^	GLP^3^	BLP^4^		CP^6^	Sex^7^	CP × Sex
*Firmicutes*	21.218	26.178	50.059	25.597	5.936	0.248	0.421	0.230
*Bacteroidetes*	27.091	28.697	17.117	24.686	2.015	0.098	0.268	0.468
*Proteobacteria*	19.992^a^	6.7039^b^	8.8265^b^	16.676^a^	2.442	0.904	0.584	0.043*
*Fusobacteria*	15.595	15.525	11.367	14.503	2.357	0.584	0.748	0.737
*Gracilibacteria*	1.9123^a^	0.6342^b^	0.2604^b^	1.0240^ab^	0.195	0.122	0.518	0.017*
*Actinobacteria*	2.2497	4.9422	5.6000	11.176	1.080	0.038*	0.070	0.512
*Spirochaetes*	1.0922	6.4712	4.0672	1.2238	1.139	0.623	0.584	0.086
Unidentified Bacteria	10.620	8.6731	1.9294	4.7574	0.962	0.004**	0.821	0.229
*Synergistetes*	0.0332^a^	1.7022^b^	0.5048^ab^	0.1910^a^	0.214	0.239	0.129	0.031*
*Euryarchaeota*	0.0011	0.1299	0.0127	0.0058	0.031	0.377	0.339	0.288
Others	0.1962	0.3439	0.2554	0.1600	0.038	0.418	0.732	0.122

*Data are means of eight observations.*

*^1–4^GHP, gilts fed high protein diet; BHP, barrows fed high protein diet; GLP, gilts fed low protein diet; BLP, barrows fed low protein diet.*

*^5^SEM, standard error of the mean.*

*^6^CP: crude protein levels, for high protein level and low protein level.*

^7^Sex: for gilts and barrows.

*Statistically significant (*P* < 0.05); ** statistically very significant (*P* < 0.01).

*a, b: statistically significant (*P* < 0.05) by comparison between two groups.*

**TABLE 3:** Relative abundance of microbial community (Top 10, %) structure in colonic content at genus level from each group.

Index	Treatment				SEM^5^	*P*-value		
	GHP^1^	BHP^2^	GLP^3^	BLP^4^		CP^6^	Sex^7^	CP × Sex
Unidentified *Clostridiales*	1.2029	7.9914	14.546	12.967	1.984	0.029*	0.517	0.301
*Neisseria*	14.766^a^	1.3843^b^	0.8689^b^	2.7486^b^	1.457	0.041*	0.059	0.014*
Unidentified *Prevotellaceae*	16.590^a^	4.1649^b^	1.8064^b^	2.8393^b^	0.736	0.000**	0.001**	0.000**
*Terrisporobacter*	0.0010	8.7390	9.4228	5.7508	1.527	0.302	0.414	0.052
*Leptotrichia*	15.745	5.6272	6.1557	4.6449	1.526	0.095	0.067	0.170
*Lactobacillus*	0.0228	3.5656	1.2407	3.1292	0.733	0.792	0.075	0.577
*Gracilibacteria*	1.6931^a^	0.4756^b^	0.2604^b^	0.5139^b^	0.165	0.044*	0.156	0.035*
*Fusobacterium*	1.1792	6.0248	5.2115	2.6279	0.949	0.868	0.556	0.061
*Spirochaetaceae*	0.9484	4.8888	3.7192	0.9139	0.887	0.737	0.751	0.068
Unidentified Bacteria	9.6491^a^	4.0257^b^	0.3225^b^	0.9513^b^	0.685	0.000**	0.079	0.031*
Others	38.202	53.113	56.446	62.913	1.497	0.000**	0.001**	0.170

*Data are means of eight observations.*

*^1–4^GHP, gilts fed high protein diet; BHP, barrows fed high protein diet; GLP, gilts fed low protein diet; BLP, barrows fed low protein diet.*

*^5^SEM, standard error of the mean.*

*^6^CP: crude protein levels, for high protein level and low protein level.*

^7^Sex: for gilts and barrows.

*Statistically significant (*P* < 0.05); ** statistically very significant (*P* < 0.01).

*a, b: statistically significant (*P* < 0.05) by comparison between two groups.*

### Metabolome Profiles and PCA of the Main Metabolites in the Colonic Content of Barrows and Gilts Induced by Dietary Protein Levels

To reveal the effects of dietary protein levels and pig sex on intestinal metabolic profiles, LC-MS was used to analyze the metabolome of the colonic content. As shown in Supplementary Table 3, the score plot of LC-MS [electrospray ionization negative (ESI-)] data with 2,037 metabolite signals and LC-MS (ESI+) data with 3,844 metabolite signals were detected.

**FIGURE 4:**
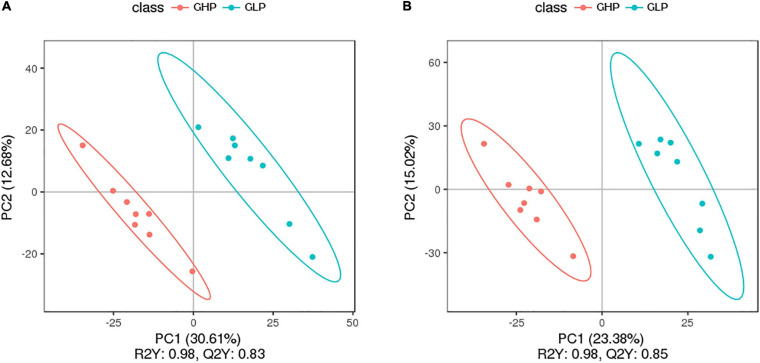
PLS-DA score plot of colonic metabolomic data from gilts fed low and high protein diets. **(A)** LC-MS (ESI-); **(B)** LC-MS (ESI+). GHP, gilts fed high protein diet;GLP, gilts fed low protein diet.

From the perspective of dietary protein levels [Supplementary Figure 2A(a–d)], the PCA results showed that dietary protein levels had a robust influence on main metabolites of pigs, especially between GHP and GLP groups, in which the metabolic communities were clustered. From the perspective of pig sex [Supplementary Figure 2B(a–d)], the PCA results showed that the main metabolites between the two pairs of BHP and GHP, and BLP and GLP groups were mixed together. Especially for barrows, there was a marked variation among samples even in the same group. In contrast, samples of gilts were more gathered. The PLS-DA score plots (Figures 4A,B) also showed that the GHP and GLP groups were well-separated, suggesting that dietary protein levels caused more significant biochemical changes in gilts compared with that in barrows. These results suggested that main metabolites between gilts and barrows and within barrows fed different dietary protein levels had no significant differences. Therefore, in this study subsequent analysis on microbial different metabolites-related results mainly focused on the experimental gilts fed the HP and LP diets.

### Identification and KEGG Analysis of Differently Abundant Metabolites in Colonic Content of Gilts Fed the High Protein and Low Protein Diets

**FIGURE 5:**
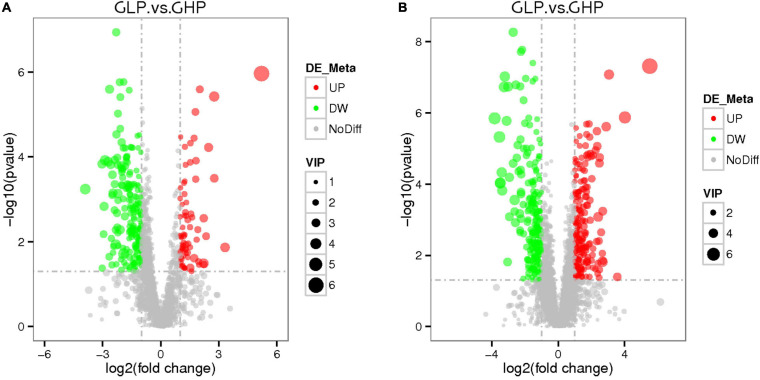
Score volcano plots of colonic metabolomic data from gilts fed low and high protein diets. **(A)** LC-MS (ESI-); **(B)** LC-MS (ESI+). GHP, gilts fed high protein diet; GLP, gilts fed low protein diet.

Furthermore, the parameters of variable importance of projection (VIP) > 1.0 and adjusted *q* < 0.05 were used to detect differentially abundant metabolites in response to different dietary protein levels in gilts. As shown in Figures 5A,B and Supplementary Table 4, compared with those in the GHP group, a total of 156 differentially abundant metabolites in LC-MS (ESI-) were identified in the GLP group, including 32 increased and 124 decreased abundant metabolites. Similarly, 278 metabolites in LC-MS (ESI+) were identified, including 126 increased and 152 decreased abundant metabolites. These results suggested that the low protein diet induced more numbers of metabolites’ abundances decrease and less numbers of metabolites’ abundances increase in all differentially abundant metabolites detected.

**FIGURE 6:**
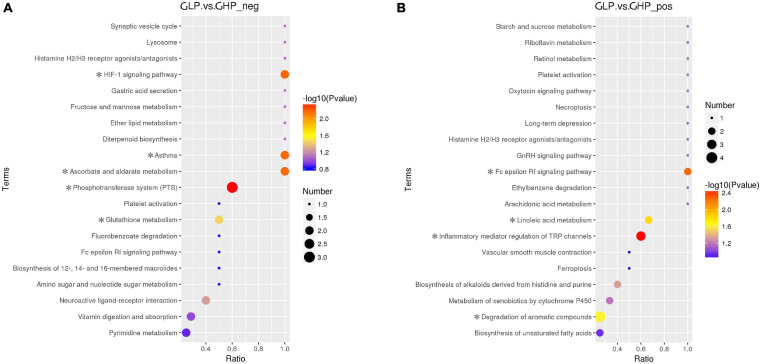
KEGG pathway enrichment of the differentially abundant metabolites between the two gilt groups. ^∗^*P* < 0.05, **(A)** LC-MS (ESI-); **(B)** LC-MS (ESI+). GHP, gilts fed high protein diet; GLP, gilts fed low protein diet.

The KEGG was used to analyze the pathways of the differentially abundant metabolites between the two gilt groups. As shown in Figures 6A,B and Supplementary Table 5, the metabolic pathways of the Phosphotransferase system (PTS), Ascorbate and aldarate metabolism, the HIF-1 signaling pathway, and Asthma and Glutathione metabolism were associated with four metabolites in LC-MS (ESI-) and were significantly affected by dietary protein levels. Inflammatory mediator regulation of TRP channels, the Fc epsilon RI signaling pathway, Linoleic acid metabolism, Degradation of aromatic compounds, and Biosynthesis of alkaloids derived from histidine and purine were associated with nine metabolites in LC-MS (ESI+) and were significantly affected by dietary protein levels. Interestingly, the metabolite vitamin C (also named ascorbic acid), was enriched and regulated the pathways of PTS, Ascorbate and aldarate metabolism, the HIF-1 signaling, and Glutathione metabolism.

### Correlation Between the Predominant Microbial Community and Differentially Abundant Metabolites in the Colonic Content of Gilts Induced by Dietary Protein Levels

There were six genus-level microbial communities whose proportions showed significant differences in response to dietary protein levels (Supplementary Table 6). Compared with the GHP group, the proportions of unidentified *Clostridiales* (*P* < 0.05) and *Terrisporobacter* (*P* < 0.01) were significantly increased in GLP and the proportions of the remaining communities, *Neisseria* (*P* < 0.05), unidentified *Prevotellaceae* (*P* = 0.00), *Gracilibacteria* (*P* < 0.05), and unidentified bacteria (*P* < 0.01), were decreased.

**FIGURE 7:**
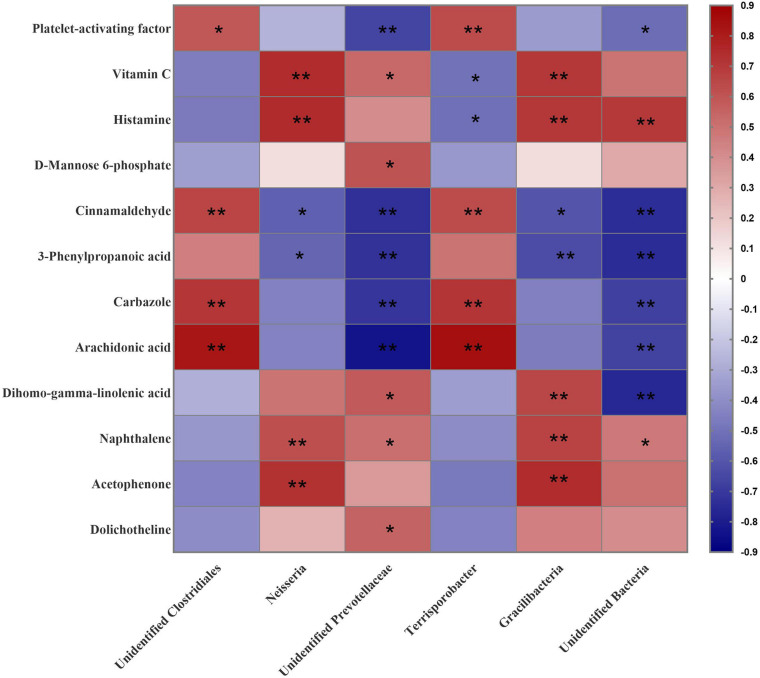
Correlation analysis between the predominant microbial community at genus level (relative abundance) and differentially abundant metabolites in the colonic content of gilts induced by different dietary protein levels. *P < 0.05; **P < 0.01.

To further reveal the crosstalk between the microbiota and the host, the six communities were selected and used to analyze the correlation with 12 changed metabolites that were enriched in above KEGG analysis. As shown in Figure 7, the proportion of unidentified *Clostridiales* was associated positively with the levels of the Platelet-activating factor (*P* < 0.05), Cinnamaldehyde (*P* < 0.01), Carbazole (*P* < 0.01), and Arachidonic acid (*P* < 0.01). *Neisseria* was associated positively with Vitamin C (*P* < 0.01), Histamine (*P* < 0.01), Naphthalene (*P* < 0.01), and Acetophenone (*P* < 0.01), and negatively with Cinnamaldehyde (*P* < 0.05) and 3-Phenylpropanoic acid (*P* < 0.05). Unidentified *Prevotellaceae* was associated positively with Vitamin C (*P* < 0.05), D-Mannose 6-phosphate (*P* < 0.05), Dihomo-gamma-linolenic acid (*P* < 0.05), Naphthalene (*P* < 0.05) and Dolichotheline (*P* < 0.05), but negatively with Platelet-activating factor (*P* < 0.01), Cinnamaldehyde (*P* < 0.01), 3-Phenylpropanoic acid (*P* < 0.01), Carbazole (*P* < 0.01), and Arachidonic acid (*P* < 0.01). The proportion of *Terrisporobacter* was associated positively with Platelet-activating factor (*P* < 0.01), Cinnamaldehyde (*P* < 0.01), Carbazole (*P* < 0.01), and Arachidonic acid (*P* < 0.01), but negatively with Vitamin C (*P* < 0.05) and Histamine (*P* < 0.01). *Gracilibacteria* were associated positively with Vitamin C (*P* < 0.01), Histamine (*P* < 0.01), Dihomo-gamma-linolenic acid (*P* < 0.01), Naphthalene (*P* < 0.01), and Acetophenone (*P* < 0.01), but negatively with Cinnamaldehyde (*P* < 0.05) and 3-Phenylpropanoic acid (*P* < 0.01). Unidentified bacteria were associated positively with Histamine (*P* < 0.01) and Naphthalene (*P* < 0.05) but negatively with Platelet-activating factor (*P* < 0.05), Cinnamaldehyde (*P* < 0.01), 3-Phenylpropanoic acid (*P* < 0.01), Carbazole (*P* < 0.01), Arachidonic acid (*P* < 0.01), and Dihomo-gamma-linolenic acid (*P* < 0.01).

## Discussion

Dietary protein intake is essential for the health and growth of humans and animals. However, inappropriate amino acid composition and protein content consumption, including both excess and deficiency, has adverse effects on the body, especially the intestinal microbiota, which is very sensible to subtle environmental changes. Previous studies reported that dietary protein levels had no significant effects on microbiota diversity; they did alter microbial composition in the hindgut of growing-finishing pigs (Zhou et al., 2016; Fan et al., 2017; Chen J. et al., 2018; González-Prendes et al., 2019; Zhao et al., 2020). However, our results revealed that the low protein diet not only changed the microbiota composition, but also increased microbial species and improved microbial diversity and richness in growing-finishing pigs. These inconsistent results might be related to the dietary protein content used and the reduction levels. The gut microbiota was also demonstrated to be affected by sampling different intestinal segments. Dietary protein levels changed the ileal microbiota diversity rather than that in feces in weaned piglets (Pollock et al., 2019). Moderate lowering of the dietary protein concentration also improved the α-diversity of that intestinal microbial community in growing pigs (Peng et al., 2017; Chen X. et al., 2018). However, an excessive protein decrease, i.e., a very low protein intake, was detrimental to the microbiota structure (Shi et al., 2019; Spring et al., 2020). Therefore, changes in microbiota upon dietary protein in pigs are dose- and site-dependent (Zhang et al., 2020). Our results demonstrated that the dietary protein content could affect the intestinal microbiota diversity in pigs at the growing-finishing stage, and suggested that the low protein diet formulated in the present study was appropriate and helpful to increase intestinal microbial diversity and species in growing-finishing pigs.

In terms of intestinal microbiota composition, our results demonstrated the four main phyla *Firmicutes*, *Bacteroidetes*, *Proteobacteria*, and *Fusobacteria* in all groups accounted for over 77% of all bacteria phyla. The former three phyla were also demonstrated to be the most abundant bacterial communities in the feces of piglets (Shili et al., 2020). We found the proportion of *Actinobacteria* increased when the dietary protein level was lower. Although the role of *Actinobacteria* in the intestine is unclear, which are considered probiotics and could produce biological active substances with anti-inflammatory and antibacterial properties (Demain and Sanchez, 2009). Our results revealed that the low protein diet mainly increased the proportion of *Clostridiales*, and decreased *Prevotellaceae*, *Neisseria* and *Gracilibacteria*. Similarly, the abundances of *Clostridiales* were decreased by feeding high protein diets in the large intestinal content of rats and in the feces of dogs (Hang et al., 2012; Liu et al., 2015). However, this was contrary to the previous results (Pieper et al., 2012; Luo et al., 2015) which showed that a low protein diet decreased the relative abundance of *Clostridium* in weaned piglets. The difference was attributed to the *Clostridiales*, which were detected in growing-finishing pigs and piglets belong to different genera, suggesting they probably played different roles. Interestingly, a previous study found that the proportion of *Prevotella* in fecal samples was lower in piglets fed with a low protein diet than in those fed a high protein diet, but was higher in the ileal digesta (Kaewtapee et al., 2017). The inconsistence on the microbiota from different intestinal sampling sections was also demonstrated by Rist et al. (2014). This variation might reflect the specific functions of bacteria in different intestinal segments. Therefore, the roles of the differentially abundant bacteria in the gut should be investigated in further studies.

Although dietary protein levels had greater influence than pig sex, our results revealed there were also the differences of microbiota diversity and communities between barrows and gilts. More interestingly, the two factors showed obvious interactions in microbial community structure. The interactions resulted in the influences of dietary protein levels or pig sex being compromised or more robust, or even offset or reversed. Specifically, at the phylum level the relative abundances of both *Proteobacteria* and *Gracilibacteria* were lower in the GLP group than in the GHP group, whereas *Proteobacteria* was higher in the BLP group than in the BHP group. *Gracilibacteria* showed no significant difference between barrow groups. By contrast, *Synergistetes* showed no significant difference between gilt groups, but was lower in the BLP group than in the BHP group. At the genus level, the relative abundances of *Neisseria*, Unidentified *Prevotellaceae*, *Gracilibateria*, and unidentified bacteria were higher in the GLP group than in the GHP group and higher in the GHP group than in the BHP group, but all four communities showed no significant differences between the BLP and BHP groups, or between the GLP and BLP groups. These interactions also suggested that the low protein diet had a greater influence on intestinal microbial communities in gilts than in barrows. Thus, the present study revealed the effects of combing two factors of pig sex and dietary protein content on the intestinal microbiota, which indicated new feeding strategies could be developed, such as designing different dietary protein concentrations according to pig sex to ensuring intestinal microbiota health. In addition, our results suggested that it is necessary to use single sex animals for studying intestinal microbiota to remove sex-related inherent differences.

Dietary interventions had major influences on the metabolic composition and abundance of the intestinal microbiome (Mitchell et al., 2020). Previous studies demonstrated the roles of dietary starch, fat/fiber (Heinritz et al., 2016; Yu M. et al., 2019) and fermented feed (Lu et al., 2019) in growing-finishing pigs. Low protein diet was speculated to increase intestinal metabolic activity by the functional prediction on gut microbial flora of pigs fed different protein content diets because genes in intestinal microorganisms at different protein levels are mainly enriched in the “metabolism pathway” (Wang D. et al., 2020). Differences of gut microbiota and its metabolites in piglets induced by protein restriction were demonstrated, and low protein diet was confirmed to decrease mainly the concentrations of biogenic amines, phenolic and indole compounds, and acetate and total SCFA (Yu et al., 2017; Hou et al., 2021). Similarly, we also found an abundant decrease of histamine, phenolic and acetate compounds, etc., in growing-finishing pigs fed low protein diet. More significantly, in the present study PCA and PLS-DA analysis demonstrated that colonic metabolites were influenced by both dietary protein levels and pig sex, in which the GHP and GLP groups had a clear separation compared with the other pairs of groups. These results indicated a low protein diet specifically shifted the metabolic profile in gilts. Interestingly, an increase in protein consumption for 10 weeks had no influences on the fecal microbiota and volatile metabolites in healthy older men (Mitchell et al., 2020). Notably, in the above study, all the participants were men rather than women. In contrast, another study on female pigs rather than male also proved that sanitary conditions affected the colonic microbiome and metabolome (te Pas et al., 2020). The differences of metabolic profile between pig sexes may largely contribute to the sex-related microbial compositions by sex hormones which had been confirmed to modulate gut microbiota (Razavi et al., 2019). Sex steroid levels have been demonstrated to be correlated with intestinal microbiota composition and diversity (Kaliannan et al., 2018; Shin et al., 2019). The possible mechanisms of sex steroids on gut micorbiota could induce the permeability of intestinal mucosal integrity (van der Giessen et al., 2019), bind to sex hormone receptors in gut (Menon et al., 2013), and regulate bile acid levels (Org et al., 2016) and immune response (Fransen et al., 2017). The present study was the first time to reveal the female-specific influences on intestinal microbial metabolome in swine.

In recent years, two types of protein metabolites produced by intestinal microbes, biogenic amines and short chain fatty acids (SCFAs), which are generated by deamination and decarboxylation, respectively, have been studied (Fan et al., 2015). However, the present study found the SCFA levels were not different between the GHP and GLP groups. In fact, previous studies demonstrated that the main SCFAs, such as acetate, propionate, and butyrate, are generated in the hindgut from carbohydrates fermentation rather than proteins. Branched chain fatty acids also belong to the SCFAs, which account for about 5–10% of total SCFAs, are produced by the microbiota exclusively from branched chain amino acids (BCAAs) (Blachier et al., 2007; Ma et al., 2010). However, our results showed the abundances of BCAAs had no significant differences between dietary protein content groups. This may be due to metabolites detected using untargeted analysis and a slight difference of BCAAs’ content between two diets.

The growth performance of the pigs in this study has been reported (Shang et al., 2020). Generally, dietary protein levels had no significant influence (*P* > 0.05) on the average daily gain (ADG) and average daily feed intake (ADFI), but low protein diet improved (*P* < 0.05) the ratio of feed and gain (F/G). In contrast, pig sex had a more obvious difference in the growth performance. Barrows showed a greater ADG (*P* = 0.00) and ADFI (*P* = 0.00), but no difference (*P* > 0.05) in F/G. These results indicated a moderate decrease of dietary protein concentration could benefit for improving feed efficiency. And this may due to the different response of the intestinal microbiota and metabolites of pigs fed different protein levels. Therefore, in this study we firstly investigated the relationship of intestinal microbiota and metabolites in barrows and gilts. Pearson’s correlation analysis showed that the relative abundances of bacteria at the genus level were closely associated with the concentrations of 12 specific metabolites. Among these metabolites, histamine showed the largest decrease. Dietary histidine was considered to involve in protein-restricted response in low protein diet-fed pigs and affected gut microbiota (Kang et al., 2020). The polyamine histamine is a well-known pro-inflammatory factor (Smuda and Bryce, 2011). Consistently, KEGG analysis also found that several inflammation-related pathways were enriched. These results suggested that the intestinal *Neisseria* and *Gracilibacteria*, which are histamine-producing bacteria, might induce inflammation by the increase of histamine concentration in female pigs fed with the higher protein diet. More evidence directly related to inflammation is needed to support the speculation in future study. In addition, an abundant decrease of other biogenic amines including tryptamine, putrescine, and cadaverine was confirmed in colonic content of adult pigs fed a low protein diet (Yu D. et al., 2019). The present study also found that the dietary protein content regulated the proportions of certain special bacteria and their metabolites, such as fatty acids, vitamin C, and platelet-activating factor, which might have subsequent influences on immune, diseases and lipid related metabolism processes. Further research examining the potential roles of these different metabolites may be more significant to our understanding the function of microbiota induced by dietary protein change and its interaction with host health.

## Conclusion

Combined intestinal microbiome and metabolome analysis demonstrated that the dietary protein content selectively altered the gut microbial diversity, composition, and metabolic profiles in the colon of growing-finishing pigs. Decreasing dietary protein level with the supplement of essential amino acids improved feed efficiency of experimental pigs, increased the intestinal microbial species and diversity. The influences of dietary protein on the microbiota composition were significantly different between barrows and gilts. In particular, the concentrations of intestinal metabolites in gilts, including histamine, fatty acids, vitamin C, and platelet-activating factor, which involve amino acids, immune, and inflammation related metabolism, were altered by changing the dietary protein levels. These results suggested that decreasing dietary protein contents changed the intestinal microbiota in growing-finishing pigs, which selectively affected the intestinal metabolite profiles in gilts.

## Data Availability Statement

The datasets presented in this study can be found in online repositories. The names of the repository/repositories and accession number(s) can be found below: https://www.ncbi.nlm.nih.gov/, PRJNA738339.

## Author Contributions

ZX was the Principal Investigator of the relevant project (2021C02007). XT designed the study, in charge of this study and was the Principal Investigator of the relevant project (2018C02035), and wrote the manuscript. BD designed this study together and joined in part of this study. QY performed animal feeding experiment. XM and JW joined in part of this study. All authors were variously involved in completion of this manuscript.

## Conflict of Interest

The authors declare that the research was conducted in the absence of any commercial or financial relationships that could be construed as a potential conflict of interest.

## Publisher’s Note

All claims expressed in this article are solely those of the authors and do not necessarily represent those of their affiliated organizations, or those of the publisher, the editors and the reviewers. Any product that may be evaluated in this article, or claim that may be made by its manufacturer, is not guaranteed or endorsed by the publisher.

## Footnotes

1
https://www.mzcloud.org/

2
http://www.chemspider.com/

3
http://www.genome.jp/kegg/

## Supplementary Material

Supplementary Figure 1 Relative abundance of microbial community (Top 10) structure in the colonic content at Phylum level.

Supplementary Figure 2 PCA score plot of colonic metabolomic data for barrows and gilts fed low and high protein diets. **(A)** Effects of dietary protein levels **(a)**, BHP-BLP (ESI-); **(b)**, BHP-BLP (ESI-); **(c)**, GHP-GLP (ESI-); **(d)**, GHP-GLP (ESI-); **(B)** Effects of pig sex **(a)** BHP-GHP (ESI-); **(b)** BHP-GHP (ESI-); **(c)** BLP-GLP (ESI-); **(d)** BLP-GLP (ESI-).

Supplementary Table 1 The compositions and nutrition levels of the experimental diets (Dry matter basis, %).

Supplementary Table 2 Raw reads and selected effective sequences in each sample.

Supplementary Table 3 All metabolites detected for barrows and gilts fed low and high protein diets.

Supplementary Table 4 Differentially abundant metabolites between GHP and GLP.

Supplementary Table 5 All KEGG pathways enriched between GHP and GLP.

Supplementary Table 6 Comparison of the relative abundance (Top 10, %) of microbial community structure in colonic content at genus level between GHP and GLP.
