# Supplementary figures and images for: Low Crude Protein Diet Affects the Intestinal Microbiome and Metabolome Differently in Barrows and Gilts
Source: Front Microbiol. 2021 Aug 20;12:717727. doi: 10.3389/fmicb.2021.717727 (PMC8417834; doi:10.3389/fmicb.2021.717727)

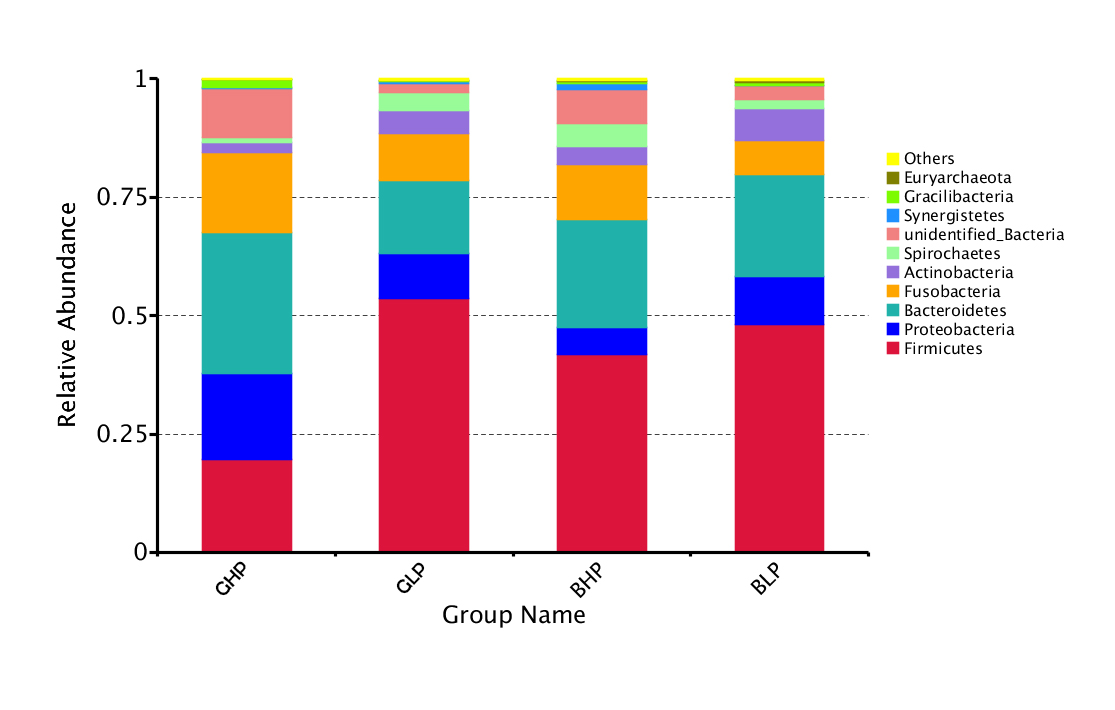

Supplement: Supplementary Figure 1 — Relative abundance of microbial community (Top 10) structure in the colonic content at Phylum level. [file Image_1.JPEG]

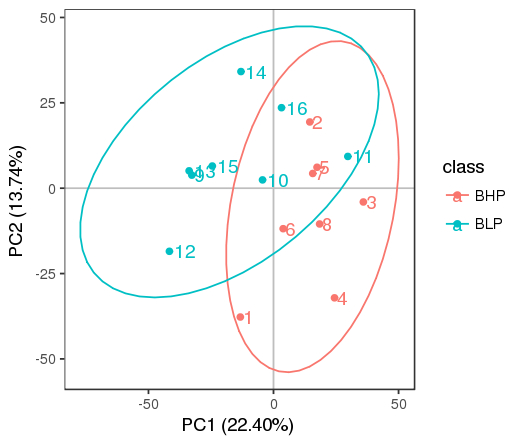

Supplement: Supplementary file 2 [file Image_2.jpg]

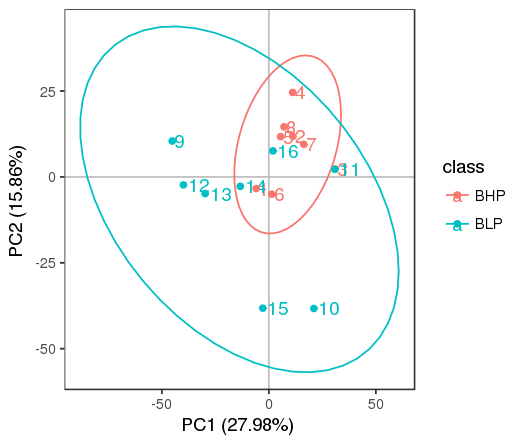

Supplement: Supplementary file 3 [file Image_3.jpg]

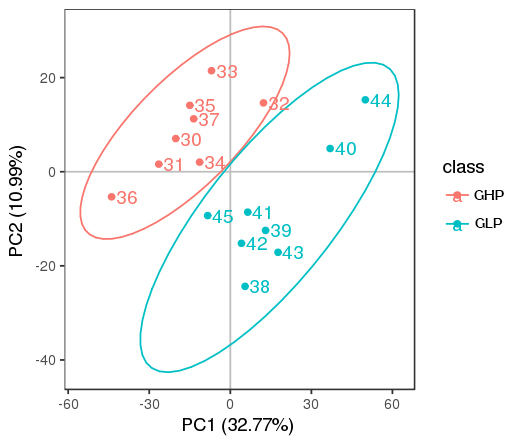

Supplement: Supplementary file 4 [file Image_4.jpg]

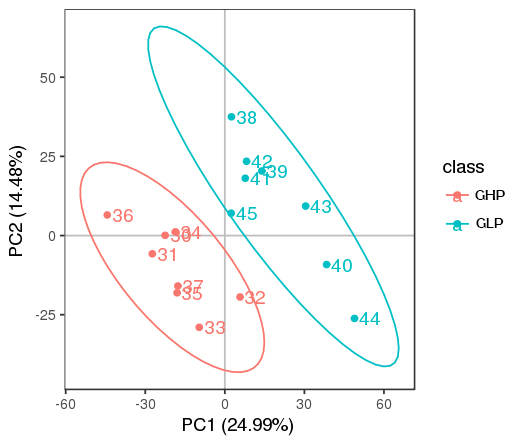

Supplement: Supplementary file 5 [file Image_5.jpg]

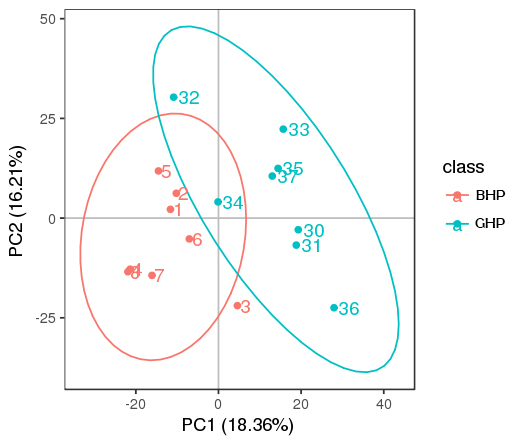

Supplement: Supplementary file 6 [file Image_6.jpg]

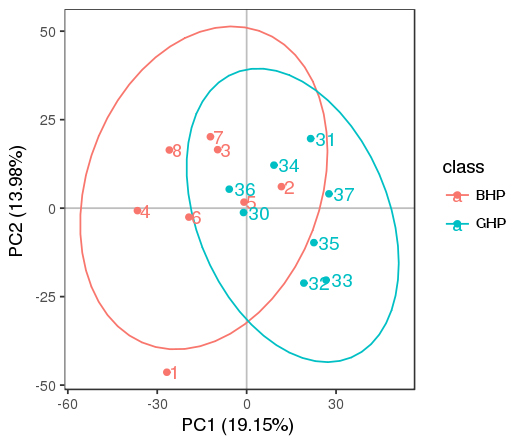

Supplement: Supplementary file 7 [file Image_7.jpg]

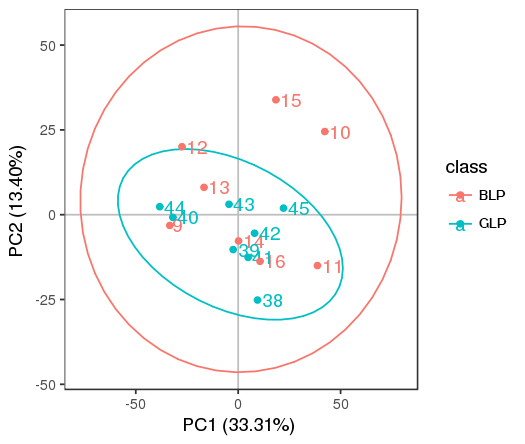

Supplement: Supplementary file 8 [file Image_8.jpg]

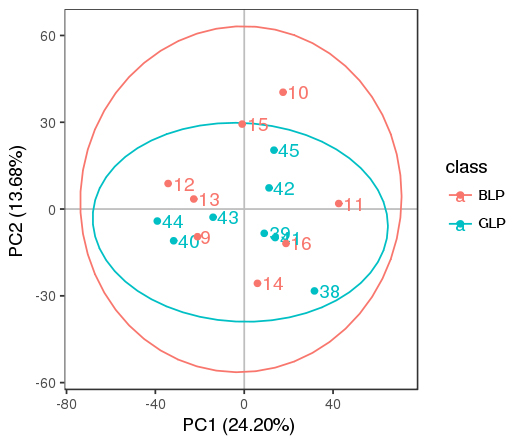

Supplement: Supplementary Figure 2 — PCA score plot of colonic metabolomic data for barrows and gilts fed low and high protein diets. (A) Effects of dietary protein levels (a), BHP-BLP (ESI-); (b), BHP-BLP (ESI-); (c), GHP-GLP (ESI-); (d), GHP-GLP (ESI-); (B) Effects of pig sex (a) BHP-GHP (ESI-); (b) BHP-GHP (ESI-); (c) BLP-GLP (ESI-); (d) BLP-GLP (ESI-). [file Image_9.jpg]
