# Supplementary material for: Low Crude Protein Diet Affects the Intestinal Microbiome and Metabolome Differently in Barrows and Gilts
Source: Front Microbiol. 2021 Aug 20;12:717727. doi: 10.3389/fmicb.2021.717727 (PMC8417834; doi:10.3389/fmicb.2021.717727)
Supplement: Supplementary Table 1 — The compositions and nutrition levels of the experimental diets (Dry matter basis, %). [file Table_1.docx]

| Items | |  | Stage Ⅰ | |  | Stage Ⅱ | |
| --- | --- | --- | --- | --- | --- | --- | --- |
|  |  |  | HP^1^ | LP^2^ |  | HP | LP |
| Ingredients (%) | | | | | | | |
| Corn | |  | 63.90 | 74.64 |  | 69.30 | 79.84 |
| Soybean meal | |  | 21.00 | 9.83 |  | 15.40 | 4.40 |
| Wheat bran | |  | 6.00 | 6.00 |  | 6.00 | 6.00 |
| Canola meal | |  | 4.00 | 4.00 |  | 4.00 | 4.00 |
| Soybean oil | |  | 1.89 | 1.26 |  | 1.72 | 0.96 |
| Limestone | |  | 0.90 | 0.90 |  | 0.90 | 0.90 |
| Zeolite powder | |  | 0.27 | 0.64 |  | 0.53 | 1.12 |
| CaHPO_4_ | |  | 0.50 | 0.50 |  | 0.50 | 0.50 |
| NaCl | |  | 0.35 | 0.35 |  | 0.35 | 0.35 |
| Lysine | |  | 0.18 | 0.52 |  | 0.19 | 0.53 |
| Threonine | |  | 0.01 | 0.15 |  | 0.08 | 0.16 |
| Methionine | |  | - | 0.04 |  | 0.02 | 0.04 |
| Tryptophan | |  | - | 0.04 |  | 0.01 | 0.05 |
| Valine | |  | - | 0.08 |  | - | 0.08 |
| Isoleucine | |  | - | 0.05 |  | - | 0.07 |
| Premix^3. 4^ | |  | 1.00 | 1.00 |  | 1.00 | 1.00 |
| Nutrient level (%) | | | | | | | |
| Net energy (MJ/kg) | |  | 10.40 | 10.40 |  | 10.40 | 10.40 |
| Crude protein^5^ | |  | 16.85 | 13.10 |  | 15.40 | 11.45 |
| Total calcium^6^ | |  | 0.64 | 0.60 |  | 0.67 | 0.64 |
| Total phosphorus^7^ | |  | 0.46 | 0.41 |  | 0.41 | 0.39 |
| AID Amino Acid^8^ | Lys |  | 0.847 | 0.851 |  | 0.729 | 0.732 |
|  | Met |  | 0.241 | 0.237 |  | 0.219 | 0.211 |
|  | Cys |  | 0.444 | 0.311 |  | 0.378 | 0.247 |
|  | Thr |  | 0.521 | 0.518 |  | 0.460 | 0.460 |
|  | Try |  | 0.164 | 0.149 |  | 0.136 | 0.132 |
|  | Arg |  | 0.937 | 0.646 |  | 0.791 | 0.504 |
|  | His |  | 0.406 | 0.306 |  | 0.356 | 0.258 |
|  | Ile |  | 0.573 | 0.454 |  | 0.489 | 0.392 |
|  | Leu |  | 1.265 | 1.025 |  | 1.145 | 0.908 |
|  | Phe |  | 0.695 | 0.511 |  | 0.603 | 0.421 |
|  | Tyr |  | 0.467 | 0.340 |  | 0.404 | 0.279 |
|  | Val |  | 0.624 | 0.554 |  | 0.550 | 0.483 |

Table S1. The compositions and nutrition levels of experimental diets (Dry matter basis, %)

^1, 2^ HP, high protein; LP, low protein.

-: No addition.

^3^ Supplied per kg of diet (stage Ⅰ): Fe, 60 mg; Cu, 40 mg; Zn, 60 mg; Mn, 30 mg; Se, 0.40 mg; I, 0.4 mg; Vitamin A, 10000 IU; Vitamin D_3_, 110 IU; Vitamin E, 5.4 IU; Vitamin K, 1 mg; Vitamin B_1_, 2 mg; Vitamin B_2_, 5 mg; Nicotinic acid, 20 mg; Pantothenic acid, 15 mg; Vitamin B_6_, 3 mg; Biotin, 0.1 mg; Folic acid, 1 mg; Choline chloride, 150 mg.

^4^ Supplied per kg of diet(stage Ⅱ): Fe, 30 mg; Cu, 20 mg; Zn, 30 mg; Mn, 20 mg; Se, 0.30 mg; I, 0.2 mg; Vitamin A, 5000 IU; Vitamin D_3_, 110 IU; Vitamin E, 5.4 IU; Vitamin K, 1 mg; Vitamin B_1_, 2 mg; Vitamin B_2_, 5 mg; Nicotinic acid, 2 0mg; Pantothenic acid, 15 mg; Vitamin B_6_, 3 mg; Biotin, 0.1 mg; Folic acid, 1 mg; Choline chloride, 80 mg.

^5-7^ CP, Total calcium, Total phosphorus, were measured values, others were calculated values.

^8^ SID Amino Acid, Standardized ileal digestible basis.
