# Supplementary material for: Low Crude Protein Diet Affects the Intestinal Microbiome and Metabolome Differently in Barrows and Gilts
Source: Front Microbiol. 2021 Aug 20;12:717727. doi: 10.3389/fmicb.2021.717727 (PMC8417834; doi:10.3389/fmicb.2021.717727)
Supplement: Supplementary Table 2 — Raw reads and selected effective sequences in each sample. [file Table_2.docx]

Table S2. Raw reads and selected effective sequences in each sample.

| Treatments | Sample name | Raw Reads | Clean Reads | Base(nt) | Avglen (nt) | GC% | Q20 % | Effective% |
| --- | --- | --- | --- | --- | --- | --- | --- | --- |
| GHP | GHP1 | 83337 | 77748 | 31806806 | 409 | 70.54 | 50.42 | 93.29 |
|  | GHP2 | 87746 | 80143 | 32557740 | 406 | 70.96 | 50.29 | 91.34 |
|  | GHP3 | 78720 | 72457 | 29495761 | 407 | 72.22 | 50.77 | 92.04 |
|  | GHP4 | 78983 | 75392 | 30675807 | 406 | 70.16 | 50.79 | 95.45 |
|  | GHP5 | 87664 | 83740 | 34087726 | 407 | 71.43 | 50.71 | 95.52 |
|  | GHP6 | 58963 | 57246 | 23446302 | 409 | 70.67 | 51.27 | 97.09 |
|  | GHP7 | 73935 | 71353 | 29207347 | 409 | 71.93 | 51.06 | 96.51 |
|  | GHP8 | 81436 | 78437 | 31973995 | 407 | 71.46 | 50.45 | 96.32 |
| GLP | GLP1 | 85841 | 80170 | 32532463 | 405 | 77.88 | 52.75 | 93.39 |
|  | GLP2 | 87351 | 80272 | 32713716 | 407 | 79.81 | 52.25 | 91.9 |
|  | GLP3 | 88907 | 80145 | 33291746 | 415 | 81.28 | 51.41 | 90.14 |
|  | GLP4 | 87896 | 80228 | 33153865 | 413 | 82.04 | 50.87 | 91.28 |
|  | GLP5 | 99254 | 97062 | 39730648 | 409 | 82.17 | 52.67 | 97.79 |
|  | GLP6 | 87474 | 80317 | 32828452 | 408 | 77.13 | 52.79 | 91.82 |
|  | GLP7 | 83967 | 80166 | 32572074 | 406 | 77.37 | 53.29 | 95.47 |
|  | GLP8 | 87188 | 80084 | 32912765 | 410 | 79.59 | 51.96 | 91.85 |
| BHP | BHP1 | 85617 | 80084 | 32512644 | 405 | 79.42 | 52.56 | 93.54 |
|  | BHP2 | 82742 | 80113 | 32731414 | 408 | 80.69 | 52.23 | 96.82 |
|  | BHP3 | 88381 | 80143 | 33094162 | 412 | 79.83 | 51.61 | 90.68 |
|  | BHP4 | 84342 | 80163 | 32727227 | 408 | 79.07 | 52.64 | 95.05 |
|  | BHP5 | 85002 | 80036 | 33078946 | 413 | 80.43 | 51.76 | 94.16 |
|  | BHP6 | 85911 | 80265 | 33124414 | 412 | 79.64 | 51.6 | 93.43 |
|  | BHP7 | 85818 | 80112 | 33204254 | 414 | 80.93 | 51.14 | 93.35 |
|  | BHP8 | 85014 | 80245 | 33109645 | 412 | 80.45 | 50.12 | 94.39 |
| BLP | BLP1 | 68666 | 66048 | 26818070 | 406 | 78.91 | 52.82 | 96.19 |
|  | BLP2 | 80294 | 75468 | 30758310 | 407 | 80.35 | 52.64 | 93.99 |
|  | BLP3 | 81443 | 73688 | 30067702 | 408 | 80.23 | 52.03 | 90.48 |
|  | BLP4 | 87053 | 80172 | 32817453 | 409 | 81.76 | 52.67 | 92.1 |
|  | BLP5 | 99181 | 95801 | 39376759 | 411 | 81.46 | 52.34 | 96.59 |
|  | BLP6 | 88089 | 80179 | 33048435 | 412 | 82 | 51.03 | 91.02 |
|  | BLP7 | 56426 | 53200 | 21587732 | 405 | 81.1 | 52.58 | 94.28 |
|  | BLP8 | 85816 | 83424 | 34429156 | 412 | 82.57 | 51.2 | 97.21 |

GHP, gilts fed high protein diet; GLP, gilts fed low protein diet; BHP, barrows fed high protein diet; BLP, barrows fed low protein diet;

Raw reads, sequences removing low qualititie;

Clean reads, sequences that was used to subsequent analysis by filtering out chimera sequences; Base, base amounts of Clean reads;

AvgLen, average length of Clean reads;

Q20,Clean reads, the amount of bases with Phred values greater than 20 represents a percentage of base amounts of Clean reads, the base error rate < 0.1%;

GC (%) The amounts of bases G and C represents percentage of the total base number of Clean reads;

Effective (%), the numbers of Clean reads represents percentage of the total number of Raw reads.
