# Supplementary material for: Low Crude Protein Diet Affects the Intestinal Microbiome and Metabolome Differently in Barrows and Gilts
Source: Front Microbiol. 2021 Aug 20;12:717727. doi: 10.3389/fmicb.2021.717727 (PMC8417834; doi:10.3389/fmicb.2021.717727)
Supplement: Supplementary Table 6 — Comparison of the relative abundance (Top 10, %) of microbial community structure in colonic content at genus level between GHP and GLP. [file Table_6.docx]

Table S6 Comparison of the relative abundance (Top 10, %) of microbial community structure in colonic content at genus level between GHP and GLP

| Index |  | GHP^1^ |  | GLP^2^ |  | SEM^3^ | *P*-Value |
| --- | --- | --- | --- | --- | --- | --- | --- |
| Unidentified *Clostridiales* |  | 1.2029 |  | 14.546 |  | 3.190 | 0.017^*^ |
| *Neisseria* |  | 14.766 |  | 0.8689 |  | 3.154 | 0.020^*^ |
| Unidentified *Prevotellaceae* |  | 16.590 |  | 1.8064 |  | 2.268 | 0.000^**^ |
| *Terrisporobacte*r |  | 0.0010 |  | 9.4228 |  | 2.217 | 0.008^**^ |
| *Leptotrichia* |  | 15.745 |  | 6.1557 |  | 2.773 | 0.059 |
| *Lactobacillus* |  | 0.0228 |  | 1.2407 |  | 0.303 | 0.074 |
| *Gracilibacteria* |  | 1.6931 |  | 0.2604 |  | 0.316 | 0.014^*^ |
| *Fusobacterium* |  | 1.1792 |  | 5.2115 |  | 1.480 | 0.267 |
| *Spirochaetaceae* |  | 0.9484 |  | 3.7192 |  | 1.348 | 0.393 |
| Unidentified Bacteria |  | 9.6491 |  | 0.3225 |  | 1.563 | 0.000^**^ |
| Others |  | 38.202 |  | 56.446 |  | 3.212 | 0.002^**^ |

Notes: Data are means of eight observations.

^1,2^ GHP, gilts fed high protein diet; GLP, gilts fed low protein diet.

^3^ SEM: Standard error of the mean.

* statistically significant (*P* < 0.05); ** statistically very significant (*P* < 0.01).
